# Designing and conducting tabletop exercises to assess public health preparedness for manmade and naturally occurring biological threats

**DOI:** 10.1186/1471-2458-7-92

**Published:** 2007-05-29

**Authors:** David J Dausey, James W Buehler, Nicole Lurie

**Affiliations:** 1 RAND Corporation, Pittsburgh, Pennsylvania, USA; 2 Department of Psychiatry, University of Pittsburgh School of Medicine, Pittsburgh, USA; 3 H. John Heinz School of Public Policy and Management, Carnegie Mellon University, Pittsburgh, USA; 4 Rollins School of Public Health, Emory University, Atlanta, Georgia, USA; 5 Division of Public Health, Georgia Department of Human Resources, Atlanta, Georgia, USA; 6 RAND Corporation, Arlington, Virginia, USA

## Abstract

**Background:**

Since 2001, state and local health departments in the United States (US) have accelerated efforts to prepare for high-impact public health emergencies. One component of these activities has been the development and conduct of exercise programs to assess capabilities, train staff and build relationships. This paper summarizes lessons learned from tabletop exercises about public health emergency preparedness and about the process of developing, conducting, and evaluating them.

**Methods:**

We developed, conducted, and evaluated 31 tabletop exercises in partnership with state and local health departments throughout the US from 2003 to 2006. Participant self evaluations, after action reports, and tabletop exercise evaluation forms were used to identify aspects of the exercises themselves, as well as public health emergency responses that participants found more or less challenging, and to highlight lessons learned about tabletop exercise design.

**Results:**

Designing the exercises involved substantial collaboration with representatives from participating health departments to assure that the scenarios were credible, focused attention on local preparedness needs and priorities, and were logistically feasible to implement. During execution of the exercises, nearly all health departments struggled with a common set of challenges relating to disease surveillance, epidemiologic investigations, communications, command and control, and health care surge capacity. In contrast, performance strengths were more varied across participating sites, reflecting specific attributes of individual health departments or communities, experience with actual public health emergencies, or the emphasis of prior preparedness efforts.

**Conclusion:**

The design, conduct, and evaluation of the tabletop exercises described in this report benefited from collaborative planning that involved stakeholders from participating health departments and exercise developers and facilitators from outside the participating agencies. While these exercises identified both strengths and vulnerabilities in emergency preparedness, additional work is needed to develop reliable metrics to gauge exercise performance, inform follow-up action steps, and to develop re-evaluation exercise designs that assess the impact of post-exercise interventions.

## Background

Since 2001, state and local health departments in the US have accelerated efforts to prepare for bioterrorism and other high-impact public health emergencies. These activities have been spurred by federal funding and guidance from the US Centers for Disease Control and Prevention (CDC) and the Health Resources and Services Administration (HRSA) [1-3]. Over time, the emphasis of this guidance has expanded from bioterrorism to include "terrorism and non-terrorism events, including infectious disease, environmental and occupational related emergencies" [4] as well as pandemic influenza [5].

For any locality, the rarity of major public health emergencies necessitates the use of practice-based exercises to simulate real life experiences in order to develop and improve skills and to assess response capabilities over time. The US Federal Emergency Management Agency (FEMA) describes six levels of exercises, increasing in complexity from informational seminars that minimally exercise response capacities to simulations that mimic reality and exercise participants' capacity to implement emergency response functions [6].

Intermediate in this progression is the tabletop exercise, which FEMA describes as a "facilitated group analysis of an emergency situation." As practiced in public health, there is considerable variability in how tabletop exercises are designed and conducted. Tabletop exercises may be structured discussions of evolving events or unstructured reactions to short scenarios; participants may be limited to public health staff or involve representatives from partner agencies or organizations; scenarios may range from simple to complex; and facilitation may range from being minimally directive, allowing participants to assume responsibility for managing the discussion through "role play," to highly directive, enabling the facilitator to assure that specific questions are addressed.

Recognizing the need to exercise public health emergency response, and enabled by funding and directives from CDC and HRSA, health departments throughout the US have implemented exercise programs. These exercise programs have had varying goals, including building relationships among stakeholders [7,8], training staff [9-11], and evaluating preparedness levels [12,13], and they have been used for a variety of purposes, including to identify gaps in preparedness [14], make recommendations for improving preparedness [15], and identifying variations in preparedness across health departments [16]. These exercises have involved diverse groups of stakeholders involved in public health preparedness, such as representatives from public health [17], health care [18], agriculture [19], and emergency medical services [20]. Despite the commonality of preparedness and response components across a variety of biological threats, most exercises have been designed for single use and focus on single disease, such as smallpox [21], pandemic influenza [22], or a novel virus [23].

These exercises have focused attention on the interaction between preparedness goals and exercise strategies, and have illuminated strengths and vulnerabilities in public health emergency decision making and response capacities. The increased utilization of tabletop exercises by health departments has not been accompanied by a parallel increase in knowledge sharing about lessons learned from them, either with regard to identifying common challenges that confront health departments or strategies for effective exercise design and management. Further, the literature dealing with tabletop exercises to date consists almost entirely of case studies and descriptions of a single exercise or a single disease. This paper describes lessons learned by public health researchers at RAND, and their collaborators, about the process of developing and conducting tabletop exercises in collaboration with state and local health departments in the US and their implications for public health emergency preparedness.

## Methods

Data for this paper come from four related projects conducted from 2003–2006. Taken together, these projects involved developing, conducting, and evaluating 31 tabletop exercises with state and local health departments of different sizes and structures in 13 different states across the northeast, south, mid-west, and west regions of the country (Table 1). Participating health departments did not incur any expenses through their involvement in these exercises other than the staff time required to participate.

**Table 1:** Descriptive Characteristics of Participating Health Departments

Region	Size of Population*	Agent Tested	Length in Hours	Facilitator Involvement**	Number of Participants
Midwest	medium	novel	8.0	moderate	22
Midwest	large	pandemic flu	6.0	active	21
Midwest	medium	smallpox	3.0	moderate	17
Northeast	large	smallpox	2.0	limited	5
Northeast	medium	plague	3.0	limited	20
Northeast	medium	smallpox	3.0	active	8
Northeast	medium	anthrax	3.5	active	8
Northeast	small	smallpox	3.0	limited	12
South	medium	smallpox	5.0	moderate	12
South	medium	pandemic flu	6.0	moderate	23
South	medium	pandemic flu	6.0	active	44
South	medium	smallpox	3.0	moderate	15
South	medium	smallpox	5.0	limited	14
South	medium	botulism	6.0	moderate	23
South	medium	botulism	5.0	moderate	13
South	large	botulism	2.0	moderate	18
South	small	pandemic flu	5.0	active	16
South	small	novel	8.0	moderate	20
South	large	plague	3.0	limited	13
South	small	novel	8.0	moderate	11
South	small	botulism	5.0	limited	26
South	large	smallpox	8.0	moderate	15
West	medium	smallpox	4.0	active	15
West	medium	smallpox	4.0	active	16
West	small	smallpox	4.0	active	15
West	medium	smallpox	4.0	active	17
West	medium	smallpox	4.0	active	15
West	large	smallpox	4.0	active	11
West	large	smallpox	4.0	active	23
West	medium	plague	3.0	limited	11
West	large	smallpox	4.0	active	14

*< 100,000 = small, 100,000–1,000,000 = medium, > 1,000,000 = large;

**Mild involvement-most of exercises was role played by participants, with very little intervention or direction from facilitators; Moderate involvement-most of exercises was role played or issue discussion, with the facilitator inserting additional probes and ensuring the discussion stayed on track; Active-most of the exercise was more discussion based, with facilitator asking questions or identifying issues that were subsequently discussed.

Two of these projects, one in California and the other in Georgia, involved the conduct of exercises in multiple jurisdictions in the same state. In California, the Little Hoover Commission, a bipartisan, independent state body, asked RAND to assess California's public health infrastructure. A key component of the project, described in greater detail elsewhere [16], was the development of a tabletop exercise that simulated a smallpox outbreak. This exercise was conducted in seven local health departments across California. In Georgia, RAND collaborated with the Georgia Division of Public Health and the Rollins School of Public Health at Emory University to develop, conduct, and evaluate a series of tabletop exercises focusing on different biologic agents in seven local health departments across Georgia, as well as one exercise focused at the state level.

The two remaining projects were funded by US Department of Health and Human Services (HHS) and involved the participation of multiple local health departments. The first project involved developing ten different tabletop exercise templates and formats focusing on the local public health response to bioterrorist agents. These were tested in 13 local health departments in 12 different states. The second project involved developing a tabletop exercise to examine the interface between local health departments and health care systems in a hypothetical influenza pandemic. This exercise was tested in three local health departments in different states. Greater detail on the structure for these tabletop exercises as well as the tabletop exercise templates themselves can be found elsewhere [7,17].

All exercises focused on at least one of three related objectives: training, relationship-building, and evaluation. The structure and design of the tabletop exercises varied from project to project because their objectives were somewhat different. The key domains covered are outlined in Table 2. The level of facilitator involvement varied with the exercise objectives. At one extreme, the facilitator's role was limited to introducing the exercise scenario and periodically interjecting updates. During these exercises, the participants were encouraged to lead the discussion themselves, based on their respective roles in their agency or organization. At the other extreme, the facilitator took a very active role by leading the discussion and interjecting questions or prompts. In between were exercises in which the facilitator turned the discussion over to participants but occasionally joined the discussion to request clarifications from the participants or assure that issues critical to the exercise objectives were discussed.

**Table 2:** Tabletop Exercise Design Variability

**Design Element**	**Variability**
Exercise length	• 2–8 hours
Number of participants	• 10–40 participants
Types of participants	• Public health (local and state)
	• Law enforcement and fire
	• Emergency preparedness (local and state)
	• Elected officials and policymakers
	• Health care providers and administrators
	• Emergency medical services (EMS)
Agent/disease exercised	• Plague
	• Anthrax
	• Smallpox
	• Botulism
	• Hypothetical or Novel
	• Pandemic influenza • Avian Influenza
Facilitator involvement	• Limited
	• Moderate
	• Active

Despite these differences, all of the exercises shared common elements, including: evolving hypothetical scenarios, facilitated group discussions, and some level of collective decision making by participants emphasizing the role of local health departments in recognizing and initiating a response to an emergency. The scenarios typically began with a single case report or series of case reports that heralded a nascent disease outbreak and required a public health assessment. These situations exercised the internal communication and coordination across disciplines within health departments as well as the communication and coordination with partner agencies and organizations such as health care facilities and emergency medical service agencies. Several exercises extended beyond this initial response and included scenarios that progressed days or weeks into an outbreak, requiring greater interactions between local- and state-level authorities and attention to health care surge capacity.

Every exercise concluded with a "hot wash" in which participants discussed their collective performance, identified strengths and weaknesses, and when relevant, related their performance to experience with actual outbreaks or crises. In the latter exercises, participants were prompted to develop an initial 'action plan' that addressed key vulnerabilities identified in the exercise. The facilitators subsequently generated a written "After Action Report" (AAR) that summarized the exercise experience and highlighted the observed strengths and areas for improvement. In addition, participants completed exercise evaluation forms. These consisted of a series of structured and semi-structured questions that asked participants to discuss what they learned during the exercise and to evaluate aspects of the exercise structure and conduct. For example, participants were asked to identify key gaps in preparedness that occurred during the exercise and to identify the most useful thing they learned during the tabletop exercise. The observations reported here are based on reviewing the after action reports, participant evaluations, as well as internal team discussions and consensus following the exercise debriefings.

## Results

### Common themes

The performance of health departments that participated in our tabletop exercises varied from agency to agency. However, there were consistent themes that emerged across the agencies, regardless of the structure or the biologic agent/disease discussed; nearly all agencies struggled with a common set of challenges. These challenges, summarized in Table 3 and described below, represent critical dimensions of an outbreak response.

**Table 3:** Common Challenges Observed Across Participating Health Departments

**Issue Area**	**Observed Challenges**
Surveillance and investigation	• Notification of non-hospital health care providers
	• Level and type of staff involvement
	• Laboratory sample handling and processing
Communications	• Reactive, passive media contacts
	• Reaching vulnerable populations
	• Communicating with response partners
Command and control	• Full implementation of ICS and EOC
	• Handoffs between local and state
	• Expectations regarding the CDC
Medical surge capacity	• Concrete planning
	• Accurately counting staff
	• Recruiting, training, and mobilizing volunteers

#### Surveillance and investigation

Many local health departments did not have a structured process for notifying or soliciting case reports from health care providers in the community other than those in hospitals, largely because they did not have reliable contact information for private providers or a sure means to reach them rapidly. In most instances local health departments had good relations with staff in local hospitals (e.g., emergency department staff and infection control practitioners) but did not appear to have similar working relationships with non-hospital based practitioners.

Local public health officials were sometimes unsure about their direct role in following up with suspected ill patients and collecting and shipping clinical samples for laboratory testing. For example, there was frequently confusion around whether it was the responsibility of the local health department, the state health department, or the medical personnel at the hospital to collect laboratory samples. Once the samples were collected there was often confusion around whose responsibility it was to transport the samples, and in a few sites, local law enforcement were surprised to find out that they were the responsible party. A related issue was the ability of health departments to realistically generate enough surge capacity in their public health workforce to investigate or respond to a large event, especially one that encompassed multiple jurisdictions in the same state, thereby limiting the state health department's ability to shift manpower and resources from one jurisdiction to the next.

#### Communications

Few of the health departments in which we conducted exercises were proactive in their contacts with the media, and most waited until they were contacted by the media to begin communicating with the public. One consequence of this passive approach was that public health officials often responded defensively to early and sometimes unexpected media requests and in turn, had trouble quickly formulating an initial message to the public that was clear, informative, and alleviated anxiety.

Health departments consistently expressed uncertainty about how to effectively communicate with vulnerable or underrepresented population groups in their jurisdictions, and few had well established relationships with community leaders or organizations that could serve as messengers or communications channels to these groups. In several sites, law enforcement and EMS personnel present in exercises had greater familiarity with these groups and could help identify trusted community messengers. Further, in some communities, health departments had limited language capacities or were not sufficiently familiar with community leaders to communicate effectively with these groups.

Communicating fully and effectively with response partners (e.g., law enforcement and EMS) about their occupational health risks and personal protection was also a challenge for local public health. In particular, while public health officials were usually quick to notify response partners soon after determining an event to be significant, response partners in many cases felt that public health officials were slow to provide them with critical information about the disease in question, what their risks might be, or what actions they should take to protect themselves. As a result, response partners frequently reported feeling either left out of the process or expressed concerns about continuing to work unless the risks to them were clarified and more was done to ensure their safety on the job.

#### Command and control

The use of the National Incident Management System (NIMS) and its associated Incident Command Structure (ICS) structure is relatively new to public health. This was evident in the exercises, in that nearly all health departments had difficulties deciding if and when to implement the ICS process and in identifying the party who would serve as incident commander. Similar challenges were seen in the decision and processes related to opening an Emergency Operations Center (EOC). As a result, in many exercises, local public health officials delayed taking these steps and preferred maintaining a more informal management process. This approach was preferred even as the outbreak became progressively larger, thereby stressing these informal networks.

As outbreaks evolved, there was often a lack of clarity about whether and when local health departments should hand off control to the state health department, how responsibilities should be jointly shared between local and state authorities, and whether or when federal agencies, such as CDC, should become involved. In many of the exercises, state health departments were surprised by the level of assistance requested by their local health departments especially in the early stages of the outbreak; in other more rare examples, state health departments surprised local health departments by assuming roles and responsibilities local health departments regarded as their own. Regardless, the general consensus among local public health participants in most exercises was that CDC staff would be on the ground to help them fairly quickly, particularly in situations where bioterrorism was considered likely.

#### Medical surge capacity

Most local health departments articulated some type of plan for increasing medical surge capacity by developing alternative care sites. In most instances however, these plans were unable to hold up to even a modest amount of scrutiny during the exercise because they were superficial and lacked sufficient detail necessary for rapid implementation. Related to this issue, local health departments frequently reported that there were not enough local health care workers to manage these sites even if they could create them. For example one participant noted, "We have pop-up tents and beds to increase capacity, we just don't have pop-up people to staff them."

Even obtaining a census of available staff members turned out to be challenging as many health care participants noted that some staff would likely be double-counted, particularly nurses and security officers who might work in several institutions. Increasing staff capacity through the use of community volunteers, including retired medical personnel, while often recognized as one potential solution to staffing shortages, proved to be extremely difficult to actually implement. Public health participants universally recognized the importance of volunteers, but learned that their plans to recruit, train, and mobilize large numbers of volunteers were too vague and lacked concrete actionable steps for realistic application during a real emergency.

### Strengths and improvements over time

In nearly all exercises, we also identified a number of strengths within participating health departments. However, there was far less commonality in these strengths than we observed with the areas for improvement. Universally, we observed public health leaders and staff who were committed and struggling to 'do the right thing.' The most commonly observed strengths were strong relationships between epidemiologists and hospital infection control practitioners and between public health workers and other emergency coordinators. In some instances, prior experience with emergency planning or response, such as involvement of health departments and emergency service agencies in coastal areas in preparing for or responding to hurricanes, was associated with stronger and more facile interactions between health department officials and partner agencies.

Our exercises were conducted over a period of several years. While we did not conduct exercises with any health department more than once and did not employ an experimental design to assess changes over time, we were struck by how the performance of health departments overall improved over time. First, compared to earlier exercises, local health departments appeared far more sophisticated about their early internal processes related to notification, enhanced surveillance and large outbreak investigations. In addition, by the end of the exercise period, health departments had considered plans for surge capacity, and participating hospitals had explicit plans for cancelling emergency surgery and discharging less severely ill patients. They also appeared more acutely aware of the challenges in assuring adequate numbers of staff to provide care.

### Lessons learned about tabletop exercise design

The large number of tabletop exercises we conducted allowed us to test and compare different strategies for designing and conducting tabletop exercises. These comparisons enabled us to modify our exercises over time to build upon lessons learned from previous exercises. Below we briefly highlight five lessons we learned from this experience.

#### Exercises should be designed to achieve a specific objective

When first developing the tabletop exercises, our assumption was that a single exercise could achieve multiple objectives, such as training, relationship building, and evaluation. While these objectives are interrelated and opportunities often exist to achieve them concomitantly in the same exercise, it is critical to define the priority objective for the exercise because different objectives have different implications for exercise design. For example, if exercise participants outlined a response that was flawed or problematic, in an exercise primarily focused around the objective of training it would be appropriate for the facilitator to pause and help the participants re-think their approach. On the other hand, if the objective of the exercise is evaluation, this type of facilitator involvement can lead the participants to choose a different course of action and therefore bias the overall outcome being evaluated. Taken further, in an exercise designed to build relationships and links across disciplines or agencies, a facilitator intervention implying that a participant had made a mistake could be embarrassing or diminish that person's credibility, depending on the level of trust among participants.

#### Exercises should be as realistic as possible while remaining logistically feasible

Taken together, the optimal mix of design elements represents a balance between exercise objectives and logistic feasibility. The ideal balance is one that assures sufficient realism to provide a meaningful experience while minimizing distractions associated with the necessary artifice of exercise scenarios. Some departures from reality may be inadvertent if scenarios are developed with insufficient attention to local routines, forcing participants to sidestep usual procedures. Even seemingly minor design errors, such as using an outdated name for a hospital, or a time course for a disease that is inconsistent with its known epidemiology, can undermine the credibility of the exercise and can distract participants enough to take them out of their roles, thus disrupting the flow of the exercise.

#### Tabletop exercises should be designed around "issue areas" rather than scenarios

The desire for a realistic exercise scenario can lead to the development of tabletop exercises around scenarios rather than issue areas based on local preparedness needs and priorities. This does not ensure that the participants will address the important issue areas. A broad mix of challenges related to a given scenario must be addressed, often simultaneously, ranging from conducting epidemiologic or environmental investigations, implementing and modifying interventions as information becomes available, communicating within and across agencies, and communicating with political leaders and the public. Introducing this full set of tasks into an exercise scenario in a way that meaningfully exercises relevant capacities is unlikely to align with the exercise's objective. Moreover, different stakeholders may want to address different issue areas and may become frustrated if their expectations are not met. It is therefore important for stakeholders to agree on a limited number of priority issue areas for the exercise and then to focus the design of the scenario around these areas.

For example, one set of exercises we designed focused on pandemic influenza preparedness in local health departments. Because it was infeasible to exercise the entire pandemic plan around a single scenario and in a single exercise, we developed the scenario and then the exercise by first meeting with local stakeholders to decide on the issue areas that would be covered in the exercise. These issue areas included disease surveillance, medical surge capacity, non-pharmacological disease control, and the use of antiviral medications. The scenario was then customized to unfold to deal with each of these issue areas. Key decisions for each discussion point were then developed, as well as facilitator probes and instructions based on the specific objective of the exercise.

#### Decision making must be forced, targeted, and time delineated

If not designed or facilitated properly, an exercise can lack focus and resolution, leaving participants to wonder what exactly was accomplished during the exercise. Therefore, it is paramount that exercises are designed to focus on issue areas that require concrete decisions over a limited period of time. For example, an exercise dealing with a simulated smallpox outbreak that unfolds over time might involve a discussion period dealing with movement restrictions in which participants are asked one or more questions such as, "Should schools be closed at this point?" Participants should then be given a limited amount of time to discuss this issue and make a decision. It is the facilitator's job to keep the discussion focused on the issue area and the specific question(s) at hand and to ensure that at the end of discussion, participants have collectively made the decision(s) they were tasked to make. Exercises can be designed to have multiple such issue area discussions as the scenario unfolds.

#### Exercises should involve a limited number of participants

Depending on the goals and objectives of an exercise, an exercise can involve a narrow or wide range of potential participants. While broader inclusion would likely be more realistic, such inclusiveness needs to be weighed against the logistics of effectively managing a larger number of participants and the potential adverse effects of inclusion. For example, participants may be less comfortable discussing ideas, taking risks, or making mistakes, depending on who is in the room. Such constraints may impede the exercise process and undermine achievement of exercise objectives.

One solution to this problem would be to sequentially stage the involvement of different participants or to physically separate different groups in a way that more closely mimics actual situations. For example, some conversations that involve airing uncertainties or weighing difficult alternatives may normally involve a limited group of people, and members of that group may be more comfortable exercising such a conversation apart from colleagues from other agencies or organizations.

The disadvantage of this approach is that it is substantially more difficult logistically and it diminishes the opportunity for people from different groups to gain an understanding of one another's role and approach to problems. In those exercises where certain participants, notably law enforcement, joined the scenarios at different stages, the feedback was generally critical, and participants felt that staging participation diminished learning and team-building opportunities. Another solution is to split exercise participants into two or more groups that allow everyone to participate, often placing people at similar levels of responsibility in the same group, and to conclude the exercise with a session that brings everyone together to share what they learned.

#### Exercise design and execution may benefit from collaborative engagement of representatives from participating agencies and external developers and facilitators

The exercises described in this report represented collaborations between people familiar with local circumstances and people from outside the participating jurisdictions who had expertise in exercise design and facilitation. Because we did not test an alternative approach that exclusively involved local personnel, it is difficult to generalize from this experience about the value of engaging people from outside the participating agencies. Nonetheless, it was our impression that at certain points in the development, facilitation, and feedback steps, there was value in involving people who were not personally invested in local relationships or situations and who could offer seemingly independent advice or perspectives.

## Discussion

Tabletop exercises can provide useful insights into both strengths and vulnerabilities in public health preparedness. It is important to recognize, however, that exercise outcomes are influenced by the way they are designed and conducted. The exercises described in this report emphasized varying dimensions of public health preparedness, reflecting differences in state or local priorities for prioritizing exercise objectives. For example, some emphasized the early response to initial reports of suspect illness while others emphasized management of surges in demand for health care services that are likely to occur later emergency scenarios. Given the intellectual and emotional demands of participation in an exercise, participants (and facilitators) may be less energetic during later rather than earlier stages of an exercise scenario, affecting perceived capacity to execute different elements of a response. Potential gaps between observed and actual preparedness should be considered in interpreting after-action reports and evaluating exercises themselves.

The utility of tabletop exercises as tools to identify areas for improvement and make improvements on these problems is still evolving. Our ability to evaluate exercise performance is hampered by the lack of an evidence base about what constitutes optimal performance and by the lack of standards for assessing public health preparedness. There is a need to move beyond qualitative performance measures to ones that are quantifiable and can be measured over time. These quantifiable measures can range from simple checklists to Likert rating scales to scorecards. For example, in a series of our exercises we used checklists to assess the performance of health departments related to surveillance, risk communication and other functions.

One fairly consistent observation was that health departments identified gaps that had been identified in prior exercises or actual experience, but had not yet been addressed. Reasons for this included lack of time, and lack of knowledge about how to make change. We now conclude exercises by having health departments prioritize the challenges observed during the exercise and then have them develop initial action plans related to up to three priority items.

There are important limitations to our work and its interpretation that must be recognized. First, the nature of our exercises changed over time on a number of important dimensions, including the scenario, priority objectives, facilitation, exercise designers and facilitators, and attention to beginning an action plan after the hot wash. As a result of this variation, we are unable to provide a numerical tabulation of the numbers of health departments that struggled with each gap or displayed given strengths. Second, because we did not employ a methodology that could conclusively assess change over time, we cannot be certain that the improvements we identified were truly reflective of improvement, and not due to the inclusion of more sophisticated health departments in the latter part of our exercise period. We doubt this is the case, however, given the national emphasis on preparedness and planning and the ways in which health departments participating in later years qualitatively described their improvement. Furthermore, similar observations regarding improvements in public health preparedness during the same time period have recently been reported by others [24,25]. We also cannot asses the potential influences that external events (e.g., hurricanes, outbreaks) may have had on health departments during the time period of our work, but it is noteworthy that all exercises were concluded before Hurricane Katrina struck. In addition, our exercises were not conducted in a random sample of health departments, and the findings may not be generalizable to all health departments. Finally, as discussed above, the evidence base for determining best practices in the design and conduct of exercises is extremely thin. We share our experience in the hope that it will help others, but do not propose that our recommendations constitute best, proven practices.

## Conclusion

Developing, conducting, and evaluating tabletop exercises requires considerable planning and the perspectives of a variety of stakeholders. While these tabletop exercises identified both strengths and vulnerabilities in emergency preparedness, additional work is needed to develop reliable metrics to gauge exercise performance, inform follow-up action steps, and to develop re-evaluation exercise designs that assess the impact of post-exercise interventions.

## Competing interests

The author(s) declare that they have no competing interests.

## Authors' contributions

The manuscript was collaboratively written by all of the authors. All of the authors have read and approved the final version of the manuscript.

## Pre-publication history

The pre-publication history for this paper can be accessed here:


